# Contribution of TMS and TMS-EEG to the Understanding of Mechanisms Underlying Physiological Brain Aging

**DOI:** 10.3390/brainsci11030405

**Published:** 2021-03-22

**Authors:** Andrea Guerra, Lorenzo Rocchi, Alberto Grego, Francesca Berardi, Concetta Luisi, Florinda Ferreri

**Affiliations:** 1 IRCCS Neuromed, 86077 Pozzilli (IS), Italy; andrea.guerra@uniroma1.it; 2 Department of Clinical and Movements Neurosciences, UCL Queen Square Institute of Neurology, University College London, London WC1N 3BG, UK; l.rocchi@ucl.ac.uk; 3 Department of Medical Sciences and Public Health, University of Cagliari, 09124 Cagliari, Italy; 4 Department of Neuroscience, University of Padua, 35122 Padua, Italy; alberto.grego@aopd.veneto.it (A.G.); francesca.berardi@unipd.it (F.B.); concetta.luisi@unipd.it (C.L.); 5 Department of Clinical Neurophysiology, Kuopio University Hospital, University of Eastern Finland, 70210 Kuopio, Finland

**Keywords:** aging, transcranial magnetic stimulation, EEG, excitability, connectivity, plasticity

## Abstract

In the human brain, aging is characterized by progressive neuronal loss, leading to disruption of synapses and to a degree of failure in neurotransmission. However, there is increasing evidence to support the notion that the aged brain has a remarkable ability to reorganize itself, with the aim of preserving its physiological activity. It is important to develop objective markers able to characterize the biological processes underlying brain aging in the intact human, and to distinguish them from brain degeneration associated with many neurological diseases. Transcranial magnetic stimulation (TMS), coupled with electromyography or electroencephalography (EEG), is particularly suited to this aim, due to the functional nature of the information provided, and thanks to the ease with which it can be integrated with behavioral manipulation. In this review, we aimed to provide up to date information about the role of TMS and TMS-EEG in the investigation of brain aging. In particular, we focused on data about cortical excitability, connectivity and plasticity, obtained by using readouts such as motor evoked potentials and transcranial evoked potentials. Overall, findings in the literature support an important potential contribution of TMS to the understanding of the mechanisms underlying normal brain aging. Further studies are needed to expand the current body of information and to assess the applicability of TMS findings in the clinical setting.

## 1. Introduction

Physiological aging is a finely controlled process which entails biological alterations at different dimensional scales, from molecules, to cells and integrated systems [1,2]. Some of these changes likely represent the undesirable outcome of exposure to stressors [3], whereas others are physiological and might subtend the attempt of the organism to maintain its function. The latter is particularly true for the central nervous system, whose functional remodeling during aging supports the preservation of activity performance in daily tasks [4]. Identifying specific patterns of neuronal activity linked to aging is a major challenge in neuroscience. In the motor system, for instance, neuromuscular degeneration, alterations in motoneuronal properties, changes in the activity and connectivity of multiple cortical circuits and modifications in the efficiency of synaptic plasticity mechanisms can be observed in older people [5,6,7,8,9]. Some of these abnormalities overlap those occurring in the early stages of patients with neurodegenerative diseases, including movement disorders or dementia [10,11,12,13,14,15,16,17,18,19,20]. Thus, understanding the various changes underlying physiological aging may help in discriminating between normal and pathological conditions. Neurophysiological techniques, in particular transcranial magnetic stimulation (TMS) and TMS-electroencephalography (EEG) coregistration, are very useful to this aim. Indeed, they are able to assess the activity of several brain circuits with a very high-temporal resolution, thus allowing to identify even subtle changes in the functionality of mechanisms controlling movement, learning and cognitive performances [21,22]. 

In this review, we will describe changes in cortical excitability, connectivity and plasticity occurring during physiological aging in humans, as assessed by TMS and TMS-EEG studies. Since TMS allows to explore mainly neurophysiological functions of the primary motor cortex (M1), the majority of studies discussed in this article will focus on the motor system. In the first section of the review we report research providing evidence for spatially-restricted (i.e., limited to M1) neurophysiological changes during aging. Indeed, over the last decade, a large number of studies found that aging is associated with changes in global corticospinal excitability and function of different neurotransmitter systems within M1, including GABA-A-ergic, GABA-B-ergic, cholinergic and glutamatergic. In the second section we discuss age-related modifications occurring in large-scale sensorimotor networks, as investigated by TMS and TMS-EEG connectivity measures. Lastly, in the third section of the article, we review non-invasive brain stimulation (NIBS) studies which assessed possible alterations in synaptic plasticity and metaplasticity during aging. Indeed, cortical plastic changes occur throughout the normal lifespan in response to the numerous events that represent everyday experiences, and synaptic plasticity mechanisms, such as long-term potentiation (LTP) and long-term depression (LTD), must be tightly regulated to prevent saturation, which would impair learning and memory [23,24,25,26,27,28]. 

## 2. Neurophysiological Changes in Local Motor Circuits during Aging

### 2.1. TMS Studies

In the last decades, TMS has been extensively used to investigate the physiology of brain aging in a safe and non-invasive way, particularly with regards to the sensorimotor system. It is well known that a single TMS pulse results in multiple descending volleys, i.e., an early direct (D) followed by indirect (I) waves [29]. The physiological mechanisms underlying the generation of I-waves are still unclear and different hypotheses have been made, ranging from oscillating activation of corticospinal tract (CST) neurons, to reverberation of activity within interneuronal circuits and converging pyramidal output cells [30,31,32]. The motor-evoked potential (MEP) arises from the temporal summation of these descending volleys at the spinal level, but its basic characteristics (i.e., latency and amplitude) also reflect the combination of excitatory and inhibitory events occurring in a more complex synaptic network, at different levels of the motor pathway [31,33]. The easiest and most reproducible single-pulse (sp) TMS measure, used to probe the excitability of M1, is the resting motor threshold (RMT), which is defined as the stimulation intensity required to elicit MEPs in resting muscles of at least 50 μV in 5 out of 10 trials [30,34]. The RMT can be considered as an estimate of global cortical excitability, it reflects axonal excitability, likely of the intracortical elements activated by TMS, and it has been reported to have high test-retest reliability in healthy older adults, in particular when using monophasic rather than biphasic TMS pulses [35]. It is sensitive not only to cortical excitability, but also to the scalp-to-cortex distance, as this alters the amount of energy required to bring CST neurons to threshold [36]. Given that age-related cortical atrophy can increase the coil-to-cortex distance, the RMT can be used as a measure to explore brain aging [37,38]. Indeed, several studies reported an increase in RMT in older adults [39,40]; however, this has not been replicated by other authors [41,42,43]. This inconsistency can be due to the possibly different degree of individual cortical atrophy in elderly subjects and to the lack of a structural neuroimaging assessment in the various studies, which would allow a coil-to-cortex distance measurement in all participants and, accordingly, an ad-hoc RMT normalization. Another hypothesis proposed to explain the lack of consistency in age-related differences in RMT is that this variable changes in discrete stages during the life span. Supporting this idea, Shibuya and colleagues (2016) recently evaluated the RMT in 113 subjects with an age ranging from 20 to 83 years; they found that the RMT increases from age 20 to 50 and then decreases, roughly following a quadratic function [44]. Up to date, a long-term longitudinal RMT follow up is missing; this could potentially have more value, compared to cross-sectional measurements, in clarifying electrophysiological correlates of the gray matter volume loss in the aging brain.

In addition to RMT, the stimulus-response relationship may be intrinsically altered in the aged motor cortex. It is well known that the MEP amplitude increases sigmoidally with stimulation intensity, and that voluntary activation of the target muscle shifts this input-output curve to the left [45,46]. Pitcher and colleagues (2003) explored the input-output curve in young and elderly subjects and demonstrated a rightward shift in the latter group, due to the higher stimulation intensity necessary to achieve the maximum MEP. The slope of the curve and the RMT were not different between the two groups [47]. These data have been interpreted as reflecting a reduction in the amount of spinal motoneurons activated by TMS or in the stimulus-induced neuronal synchronization with aging [47,48].

Besides spTMS, more complex TMS protocols, which consist in delivering two stimuli via the same coil (paired-pulse TMS; ppTMS) [49,50] or by coupling a TMS pulse with peripheral electric stimulation [51] have been also applied in older subjects. While spTMS measures generally reflect the degree of the overall corticospinal excitability [52], these protocols allow to investigate the physiology of intracortical circuits which rely on different neurotransmitter classes [30,53,54]. More in detail, in ppTMS a conditioning stimulus (CS) precedes a test stimulus (TS) by an interstimulus interval (ISI) which varies according to the protocol used, allowing the evaluation of intra-cortical inhibitory and facilitatory circuits. Short-interval intracortical inhibition (SICI) is elicited when a subthreshold CS is followed by a suprathreshold TS with an ISI of 1–6 ms and likely reflects inhibition mediated by GABA-A receptors, while intracortical facilitation (ICF) entails longer ISIs (6–30 ms) and is likely mediated by NMDA receptors [30,49,53,55]. The investigation of ppTMS measures in physiological aging has produced inconsistent results. SICI has been reported to be either decreased [41,56,57,58,59], normal [42,43,60,61] or even increased [39,62] in elderly. Differently, ICF was found to be generally normal in the majority of [41,44,58,62], but not all [39,61], studies, suggesting intact NMDA-related glutamatergic neurotransmission in these subjects. Long-interval intracortical inhibition (LICI) is another ppTMS measure, which refers to inhibition of a test MEP using a suprathreshold CS applied 50–200 ms before, and likely depends on GABA-B receptors [53,63,64]. Similar to what observed with SICI, the effectiveness of LICI was found to be variable with aging, being either increased [39] or decreased [43,58] in different studies. Thus, ppTMS researches have not yet clarified whether GABA-ergic neurotransmission is generally preserved or impaired in physiological aging. Finally, short interval intracortical facilitation (SICF) can be elicited by a suprathreshold first stimulus followed by a second stimulus at about RMT [65,66], by an ISI compatible with I-waves period (i.e., 1.3–1.5 ms). In this protocol, the observed increase in MEP is likely due to summation of different I-waves at the M1 level and mainly reflects the activity of non-NMDA glutamatergic neurotransmission [17,31]. Clark and colleagues (2011) described a higher SICF in elderly than young subjects at ISI 1.5 ms, while the opposite result was present at ISI 2.5 ms. These apparently contrasting data confirm that physiological mechanisms underlying the first and later SICF peaks differ [17,31,67,68] and may suggest that they have different sensitivity to the aging process [69]. More recently, however, Opie and colleagues tested SICF in young and older adults in more detail and compared I-wave intervals that were optimal for I-waves summation in each group. Although they confirmed the reduction in the second SICF peak in the elderly, a similar change was also found in the other peaks, thus not corroborating the previous hypothesis and leaving the question open [70,71]. Additionally, these studies found a delayed latency of the third SICF peak with aging, which influences both plasticity induction and motor function [70,71].

Discrepancies in the effects of ppTMS protocols across studies can have several explanations. The first could be the choice of the ISI due the fact that paired-pulse protocol is associated with a relatively wide range of possible ISIs, and the timing optimal to obtain the predicted changes in MEP may be different across subjects [30]. While some of ppTMS studies have used multiple ISIs [41,42,44,58], the majority employed only one ISI for all subjects [39,43,56,57,59,60,61]. As different subjects will probably manifest optimal responses at different ISI, the outcome of future experimental studies could be improved by the use of paired-pulse curves which may decrease the variability and improve the outcome in future studies. Furthermore, concerning SICI, many studies tested this measure only using an ISI of 3 ms, which, according to some reports [72], may be influenced by SICF; however, this has not been universally confirmed [66]. Another important factor contributing to the inconsistency of ppTMS protocols is the choice of CS intensity, which can be calibrated either based on the RMT, or the active motor threshold (AMT). The intensities used vary considerably, usually ranging from 50% to 95% RMT [39,41,42,44,55,56] or from 70% to 90% AMT [73,74,75], and this variability may contribute to divergent findings in SICI studies. Another point to take into account is that ppTMS measures may have different sensitivity to coil orientation [66,76], and that this pattern of susceptibility to current direction might change according to the age of subjects. In this regard, Sale and colleagues (2016) assessed SICI in young and older adults with TMS coil in posterior-to-anterior (PA) and, then, anterior-to-posterior (AP) position. Interestingly, they found no age-related differences in SICI with PA-directed TMS, while SICI was more effective in older than young subjects using AP-directed TMS [77]. Unfortunately, both the CS and TS were delivered with the same current direction, thus making it difficult to understand which one drives the effect. A further important issue is that ppTMS measures are known to be modulated by different brain states, including motor tasks [78,79], and that this change may be dependent on age. For instance, the amount of SICI during a reaction time task was related to manual dexterity in aging subjects [57]. Moreover, while SICI increases during motor stopping in young subjects, this phenomenon does not occur in older adults [80]. Analyzing task-related changes in ppTMS measures may thus increase their sensitivity in investigating possible age-related decline in intracortical motor circuits. Overall, future studies should standardize the methodology of ppTMS to help comparisons across different age groups.

Differently from ppTMS, short-afferent inhibition (SAI) has shown more consistent alterations in the aging brain. SAI is elicited by electrical stimulation of a peripheral nerve preceding contralateral M1 TMS by an ISIs slightly longer than the latency of the N20 component of the somatosensory evoked potential. This form of inhibition likely reflects intracortical sensorimotor interaction and involves cholinergic as well as GABA-A-ergic circuits [51,81,82,83]. Although a first study found that SAI was comparable between older and younger adults [42], later studies showed decreased SAI in the elderly [40,84,85,86], which was also correlated with the age of subjects [87]. These data overall point to a progressive impairment of cholinergic activity during aging.

### 2.2. TMS-EEG Studies

TMS-EEG allows to measure the perturbation induced by a TMS pulse on cortical activity, both at the local and network level. Thanks to its high temporal resolution, TMS-EEG can provide meaningful clues on the functional properties of cerebral circuits in physiological and pathological conditions [88,89,90,91,92]. Importantly, TMS–EEG allows the assessment of cortical physiology by discerning causal interactions from pure temporal correlations [93,94]. TMS-evoked EEG responses were first measured in the late nineties [95] and, subsequently, scalp topography and possible generator sources of TMS-evoked potentials (TEPs) have been described. It is now clear that TEPs represent genuine responses of the cerebral cortex to TMS, provided that indirect sources of brain activation, such as somatosensory and auditory stimulation, are appropriately reduced or suppressed [96,97,98,99]. TEPs occurring in the first 20–40 ms after the TMS pulse most likely reflect the responses of local cortical circuits, whereas longer-latency responses involve more distributed networks [100]. TMS-EEG studies which aimed to investigate age-related modifications in M1 generally showed decreased excitability, a finding which supports the outcomes of MEP investigations [42,47,101]. However TMS-EEG yields complex results, based on the metric used for analysis [102]. For instance, the global-mean field power (GMFP), which provides a measure of the TMS-induced brain response on the whole scalp [103,104], has been shown to be smaller in the elderly, pointing towards a generalized cortical hypoexcitability. This is in agreement with analysis of single TEP peaks, which showed a decrease in the local P30 amplitude after M1 stimulation; however, an opposite trend was found in the ipsilateral prefrontal areas, reflected in an increased TEP amplitude. This has been interpreted as a true hyperexcitability of the prefrontal cortex, rather than a compensatory phenomenon to M1 hypoexcitability [102]. The result on P30 has not been confirmed by another study, which failed to identify an amplitude difference between groups, suggesting instead a decreased P30 latency in elderly subjects [105].

TMS-EEG allows the investigation of specific cortical neurotransmitter systems, with the assumption that different TEP components reflect selective activity of receptor classes. Data obtained in humans and non–human primates point to age-dependent changes in GABA-A and GABA-B receptor density and subunit composition, particularly in frontal cortices [106,107]. In particular, while GABA-ergic neurotransmission seems to become more efficient during the first years of postnatal development in the dorsolateral prefrontal cortex (DLPFC) [108], there is evidence for GABA-ergic neurons activity impairment in animal and human motor areas with aging [57,58,59,109,110,111]. Previous TMS-EEG data have found that the P30 and N45 peaks are related to GABA-A neurotransmission, whereas activity of GABA-B receptors shows a stronger link with later peaks, both in healthy and in diseased brains [82,90,100,112,113]. The amplitude of the N45 has been shown to be modulated by aging, albeit in opposite directions in two studies. It was reduced in Ferreri et al. [102], a data which supported a GABA-A-ergic neurotransmission deficit in M1 in older subjects, and increased in a following study [105]. As a further confounding factor, both P30 and N45 amplitude did not change when SICI, which measures GABA-A activity, was obtained by stimulation of DLPFC [114]. While the cause of these contradictory findings is currently unclear, it may reflect the variability observed for SICI in TMS studies in aging (see Section 2.1). Importantly, to date, no study has assessed SICI in M1 with concurrent EEG recording in older adults; this would help in better elucidating possible age-related changes in GABA-A mediated inhibition in the motor system. Only few studies used other ppTMS protocols during EEG recording in the elderly. Noda and colleagues (2017) tested NMDA receptor-mediated glutamatergic activity in the DLPFC using ICF; when comparing the results with a group of healthy controls, they found a reduction in N45 amplitude. The authors speculated that this result would reflect a functional decline in glutamatergic excitatory neurotransmission in older adults [114]. Finally, a recent study recorded TEPs during M1 LICI and demonstrated a greater inhibition of the N45 wave in older, compared to younger subjects; this may suggest that this component is particularly sensitive in detecting modulation of GABA-B-ergic inhibitory circuits which occurs during aging [105].

## 3. Neurophysiological Changes in Wide-Range Networks during Aging

### 3.1. TMS Studies

Long-range connectivity between different brain areas can be tested with TMS by delivering pulses with two different coils at specific ISIs. The most robust connectivity protocol is interhemispheric inhibition (IHI), which is obtained by conditioning a MEP with a preceding suprathreshold TMS pulse on the contralateral M1. IHI probably generated by local inhibitory interneurons within M1, which activate GABA-B receptors on principal cells [115] and are activated by interhemispheric excitatory pathways passing through the corpus callosum [116,117,118]. IHI is most pronounced at ISIs of 8–10 ms and 40–50 ms, which are referred to as short and long latency IHI, respectively (SIHI and LIHI). Most studies, but not all [119,120], testing IHI at rest, revealed that both SIHI [121,122] and LIHI [123,124,125] did not differ between older and young subjects. By contrast, there is evidence that elderly subjects have less lateralized cortical activation during various motor tasks, including hand grip [126]. In neuroimaging studies, young subjects demonstrate a decreased activity in the M1 ipsilateral to the moving hand (iM1) during task, while this pattern is cancelled or even reversed in older subjects [127]. TMS research have further explored this phenomenon and found that LIHI from the contralateral M1 (cM1) to iM1 decreased during motor tasks in older compared to young participants, suggesting a reduced inhibitory drive from cM1 to iM1 with aging [121,128]. By combining neurophysiological and neuroimaging data, the authors clarified that the reduced inhibition of the iM1 underlies a progressive involvement of this area during simple motor tasks with aging [128]. Another study demonstrated that the iM1 also contributes to motor preparation in elderly. Indeed, TMS was delivered over iM1 before an index finger movement, and this caused delayed motor responses in older, but not in young, adults [129]. Besides IHI, the interaction between the two M1 can be functionally assessed by evaluating the ipsilateral silent period (ISP), defined as the interruption of voluntary electromyographic (EMG) activity induced by suprathreshold TMS applied in the iM1. ISP is thought to be mediated by transcallosal inhibition between the stimulated and the pre-activated cM1 [116,130]. A number of studies have reported changes in ISP in older subjects, such as delayed onset and decreased depth or area [131,132,133,134]. Taken together, findings on IHI and ISP indicate a decline in interhemispheric inhibition with increasing age, with recent evidence that this decline is uniform across the lifespan [134].

In addition to the interactions between the homologous M1, IHI can be extended to a widespread inhibitory system projecting from various cortical areas, including the dorsolateral prefrontal cortex, dorsal premotor cortex and somatosensory cortex, to cM1 [118]. In this regard, one study tested whether physiological aging alters the functional connectivity between the left dorsal premotor cortex (PMd) and right M1 [124]. Paired-pulse TMS was delivered immediately before a simple left index finger movement in young and older subjects, and the data showed that only in the latter group there was a facilitatory interaction between left PMd and right M1. Moreover, the degree of modulation was associated with faster responses [124]. A more recent study combined LIHI with neuroimaging and behavioral measures to assess interhemispheric connectivity between DLPFC and cM1 during the preparation of a complex bimanual coordination task in aging [125]. Interestingly, it was found that the ability to disinhibit functional connectivity between DLPFC and cM1 was impaired in older subjects, and this alteration was paralleled by decreased bimanual performance.

Overall, based on TMS data, it may be argued that the increased activity in ipsilateral M1 and premotor regions before/during simple movements reflects the involvement of additional areas as an attempt to preserve normal motor performances despite advancing age [128,135,136]. Conversely, the altered modulatory activity of DLPFC to contralateral M1 may underlie the decline of bimanual performance in older subjects [125].

### 3.2. TMS-EEG Studies

As previously stated, recording of EEG activity during TMS provides the possibility to non-invasively and directly probe brain connectivity [89]. Particularly, it has been suggested that the first part of the TMS-evoked EEG response reflects local excitation of the stimulated cortex (see Section 2.2), whereas the spatiotemporal distribution of later TEP components over the scalp reflect the activation of distant cortical areas, either via cortico-cortical connections or projections from subcortical structures [137,138,139,140]. Recent evidence has suggested that age and neurodegeneration influence late TEP components [12,102,105,114]. Among the late TEPs described in the EEG signals evoked by M1 stimulation, the N100 is the dominant negative peak and it has been related to the GABA-B-ergic neurotransmission [100,113]. In the elderly, its scalp distribution and source activation has been demonstrated to be significantly different from younger subjects, suggesting hypoexcitability in prefrontal and premotor cortices of the stimulated hemisphere, coupled with hyperexcitability in the median anterior EEG channels [102]. The neural generators of late TEP components are not entirely clarified. In previous studies [100], it had been suggested that they could be related to the activity of reverberant cortico-cortical as well as cortico-subcortical circuits driven by GABA-B neurotransmission, and finally re-engaging the stimulated M1. Age-related differences in spatial distribution of the N100 have been confirmed by a more recent study: here, by using a LICI protocol, the authors demonstrated that the paired-pulse inhibitory effects on N100 wave are increased in older adults, thus suggesting a potentiation of pre- and post-synaptic GABA-B-mediated inhibition [105].

Previous TMS-EEG studies showed that TMS-evoked EEG signals strongly depend on the brain state at the time of stimulus delivery [94]; this can be determined by excitability of local circuits, or by the activity of diffuse neuromodulatory systems [141,142]. In agreement with this notion, features of EEG rhythms preceding a TMS pulse applied on M1 have been shown to influence MEP amplitude [143,144], and this process changes with aging [142,145]. It is known that MEP amplitude shows a degree of inter-trial variability, which depends on several factors, including fluctuations in excitability of cortical and spinal neurons [146,147] and corticospinal connectivity [148]. TMS-EEG allows to verify whether MEP amplitude variability also depends on cortico-cortical connectivity changes. In this regard, Ferreri and colleagues (2014) found that, in young subjects, MEPs are significantly larger when the ipsilateral M1-prefrontal cortex coherence in the beta−2 band and the ipsilateral M1-parietal cortex coherence in the delta band are high. However, elderly subjects showed higher M1-parietal cortex delta coherence than young participants, and this measure was unrelated to MEP size variations [145]. Since the delta rhythm may underlie functional disconnection between areas [149,150], the results of this study possibly reflect functional unbinding of M1 from the somatosensory cortices’ inhibitory control. This mechanism may be compensatory to age-related decrease in cortical excitability and motor functions [145].

## 4. Neurophysiological Changes in Plasticity and Metaplasticity Processes during Aging

Physiological aging is characterized by a weakening of different brain functions, mainly linked to neuroplasticity processes, such as learning and memory [1]. In humans, various TMS-based protocols allow the assessment of synaptic plasticity mechanisms in a non-invasive way; the most widely used are paired associative stimulation (PAS) and theta-burst stimulation (TBS). PAS is based on associative synaptic plasticity [151] and its effects probably reflect spike-timing dependent plasticity, where the precise timing of pre- and post-synaptic neurons firing is crucial for the direction of long-lasting changes. If the inter-spike interval is positive (pre- before post-synaptic action potential), LTP occurs, whereas if the interval is negative (post- before pre-synaptic action potential), LTD is elicited [152,153,154,155]. In humans, the PAS protocol implies the combination of repetitive cortical TMS and peripheral nerve stimulation [156,157]. If M1 stimulation occurs around 25 ms after the electric median nerve stimulation at the wrist (PAS_25_), MEP amplitude is increased for 30–60 min (LTP-like effects), whereas if the inter-stimulus interval is shorter, i.e., 10 ms (PAS_10_), MEP amplitude is decreased (LTD-like effects) [156,157,158]. TBS is based on evidence in animal models demonstrating that high-frequency bursts of stimuli rhythmically delivered in the theta frequency range transiently modulate hippocampal neuronal firing [159], and that LTP/LTD-like changes can be recorded by measuring changes in post-synaptic responses following the stimulation [155,160,161]. The classical TBS paradigm consists of bursts of three TMS pulses at 50 Hz, repeated at 5 Hz [162,163]. If the pattern of stimulation is intermittent (iTBS), i.e., short trains of 2 s given every 10 s, cortical excitability is enhanced and MEP amplitude increases up to 30 min after stimulation (LTP-like effects). If the pattern is continuous (cTBS), i.e., bursts given continuously for 40 s, cortical excitability is inhibited and MEP amplitude decreases for 20–60 min (LTD-like effects) [155,163,164,165,166].

A recent meta-analysis on NIBS studies in the aged population suggested that there is a general trend towards decrease in motor cortex plasticity, with a certain degree of variability between different studies and different plasticity-inducing protocols [8]. In 2008, two reports by different groups showed, for the first time, that PAS-induced LTP-like plasticity of M1 may deteriorate with physiological aging [167,168]. Müller-Dahlhaus and colleagues verified the effects of PAS in a cohort of 27 subject with variable age (range: 22–71 years) and found that the magnitude of PAS effects was negatively correlated with age, with a smaller MEP facilitation in elderly subjects [167]. A direct comparison of PAS effects between young and aged subjects was provided by Tecchio and colleagues (2008). Although it was somewhat confirmed that the long-lasting increase of M1 excitability after PAS is weaker in older than young participants, this effect was clearly driven by the female population [168]. The authors pointed to a possible impairment in intracortical excitatory network activity due to hormonal changes during menopause, a hypothesis which was confirmed in a later research [169]. In a following study, a larger number of healthy subjects was enrolled and divided in three groups based on age (young: 21–39 years, middle: 40–59 years, elderly: 60–79 years). The expected PAS-induced facilitation of MEP amplitude was observed in the young and middle groups, but not in the elderly group, further confirming the impaired LTP-like plasticity in M1 with aging [170]. Interestingly, age-related decline in response to PAS has been demonstrated to be restored by L-dopa [171], a finding that suggests that the alteration in PAS response observed in the elderly might have a functional, rather than a structural substrate. In contrast with PAS, only few studies investigated the influence of aging on M1 plasticity induced by TBS. Dickins and colleagues compared MEP amplitude changes after iTBS over the dominant M1 between 20 young (18–28 years) and older subjects (65–76 years). In contrast to PAS studies, M1 excitability increased in a comparable way between the two groups after iTBS [172]. Similar findings were also obtained in more recent research, indicating that iTBS-induced LTP-like plasticity of M1 is not affected by aging [173,174]. 

The different neurophysiological findings on age-related synaptic plasticity changes obtained using PAS and TBS may depend on the different types of plasticity mechanisms elicited by the different protocols, as described above in this section [155,157,163,175,176]. Moreover, PAS acts through a combination of sensory input and direct cortical stimulation activating the same M1 neurons, a process which reflects heterosynaptic plasticity due to sensorimotor interaction [155,157,177,178]. Conversely, TBS operates through the repetitive activation of the same synapses by M1 stimulation alone, which reflects homosynaptic plasticity mechanisms [155,163,176]. Impaired PAS-induced and normal TBS-induced effects may therefore suggest that synaptic plasticity processes are not diffusely impaired by physiological aging. Rather, the alteration is restricted to processes requiring the activation of specific intracortical circuits and/or sensorimotor interaction mechanisms.

The direction and amount of synaptic plasticity can be influenced by neuronal activity occurring immediately before or during the induction of plasticity [179,180]. These features of plasticity can be framed in the context of metaplasticity, which can be shortly defined as “the plasticity of synaptic plasticity”; this involves a wide range of mechanisms and, from a behavioral point of view, has an important role in the regulation of important brain functions, including memory and learning [27,180,181]. Metaplasticity in humans can be explored through different protocols, from priming an exogenous or endogenous plasticity-inducing protocol with NIBS, to delivering plasticity-inducing protocols with longer duration [64,73,75]. For instance, Opie and colleagues (2017) delivered iTBS 10 min after the application of sham TBS (sham TBS + iTBS), cTBS (cTBS + iTBS), or iTBS (iTBS + iTBS) in young and older participants. The results showed that, whereas priming iTBS with either cTBS or iTBS boosted M1 plasticity in young subjects, MEP facilitation after sham TBS + iTBS did not differ from iTBS + iTBS, and was even larger than cTBS + iTBS, in the older group [173]. Similar findings were also found using PAS: priming the stimulation with PAS_N20+2ms_ caused enhancement of plasticity in young but not in older subjects [182]. In another study by the same group, a visuo-motor training task was performed after facilitatory, inhibitory or sham PAS in young and older adults. While the baseline level of motor skill did not differ between sessions in young subjects, priming with PAS had a detrimental effect on skill acquisition in older ones [183]. Recent evidence suggests that the amount of NIBS-induced plasticity of M1 changes by concurrently modulating cortical gamma oscillations through transcranial alternating current stimulation (tACS), and this effect has been interpreted as reflecting gating phenomena [18,184,185,186]. However, gamma-tACS has been demonstrated to boost LTP-like plasticity induced by iTBS to a larger extent in young than older adults and, in the latter group, the effect of gamma-tACS decreased with increasing age [174]. In summary, unlike synaptic plasticity mechanisms, which seem to be altered only in part by the aging process, the existing evidence point to a higher susceptibility of metaplasticity processes by physiological aging.

## 5. Limitations, Perspectives and Conclusions

**Figure 1:**
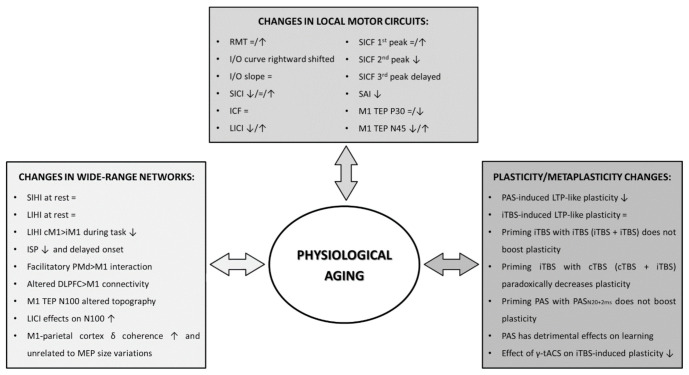
Age-related changes in local motor circuits, wide-range networks and plasticity processes RMT: resting motor threshold; I/O curve: input-output curve; SICI: short-interval intracortical inhibition; ICF: intracortical facilitation; LICI: long-interval intracortical inhibition; SICF: short-interval intracortical facilitation; SAI: short-latency afferent inhibition; M1: primary motor cortex; TEP: transcranial evoked potential; SIHI: short-latency interhemispheric inhibition; LIHI: long-latency interhemispheric inhibition; cM1: contralateral M1; iM1: ipsilateral M1; ISP: ipsilateral silent period; PMd: dorsal premotor cortex; DLPFC: dorsolateral prefrontal cortex; PAS: paired-associative stimulation; iTBS: intermittent theta burst stimulation; cTBS: continuous theta burst stimulation; tACS: transcranial alternating current stimulation.

The present review offers a summary of data obtained with TMS, either coupled with EMG or EEG, about physiological brain aging. Overall, the findings suggest that TMS can offer valuable insight into several functional derangements occurring throughout the life span, including a trend of decrease in brain excitability, altered long-range cortico-cortical connectivity and impaired associative plasticity and metaplasticity processes (Figure 1). There are, however, a number of caveats pertaining to acquisition of TMS-EMG and TMS-EEG variables, as well as their interpretation, which we deem important to discuss in this final section. The first, general issue is that, despite the relatively high number of studies investigating cortical excitability and plasticity during aging, their sample sizes are generally small. This factor would determine low statistical power and may, at least in part, explain the variability of results observed in the literature [8]. A second problem is related to the degree of cerebral atrophy which accompanies physiological aging. As this causes an increase in the distance between scalp and coil, TMS measures are necessarily affected by it [37,38]. Even the most reliable among them, such as the RMT [34], could yield limited information in a context where anatomical data are not available. Therefore, when possible, it is important to obtain structural brain information, along with neurophysiological assessment. In absence of the former, spTMS measures could still be able to track within-subject longitudinal changes, but the pathophysiological/clinical value of such follow up still need to be established. Paired-pulse TMS measures partially solve the confound represented by decreased brain volume, since they are usually calculated as ratios between conditioned and unconditioned MEPs. However, ppTMS protocols are affected by variability and reliability issues of experimental paradigms and output measures [175,187]. For instance, SICI considerably varies between individuals, even if the same CS intensities and ISIs are tested [66,188]. Therefore, to compare results across studies, it would be useful to explore a range of ISIs/CS intensities in large cohorts of subjects, so as to obtain data about maximal effects and thresholds. Moreover, since the putative circuits tested by different ppTMS protocols can interact [72], the interpretation of the effects of CS is not always straightforward. For instance, since SICI acts by suppressing I waves [189], an hypersynchronized and/or hyperexcitable state of excitatory M1 interneurons may secondarily result in SICI decrease [70,71,190]. This is possibly the case in amyotrophic lateral sclerosis [191] and Parkinson’s Disease [13,17,192,193,194]; whether a similar scenario occurs in physiological aging is not known and is probably worth exploring.

The possibility of adding simultaneous EEG recording has substantially increased the range of variables that can be tested with TMS, thus expanding the amount of information that is possible to obtain. However, the nature of TMS-EEG signals has not been completely clarified yet. For instance, the information given by TEPs obtained from M1 TMS may not completely overlap with those provided by MEPs; indeed, the latter arise from excitation of PTN and associated circuitry, whereas the former probably reflect activity of a larger ensemble of cortical cells [89,100,195]. Therefore, caution should be used when comparing conclusions drawn by the two variables, especially for ppTMS protocols, which were devised for MEPs, and are still of uncertain interpretation in the TMS-EEG setting [112,196,197]. Another issue is related to the use of TEPs to measure brain connectivity. This is usually performed by measuring the spatial distribution of specific TEP components; however, this alone does not take into account volume conduction, and has not been assessed in conjunction with more common measures of EEG connectivity [198]. In addition, TEPs, especially in their late components (N100, P180), can be contaminated by EEG responses generated by indirect brain activation due to the somatosensory and auditory stimulation intrinsic to TMS, if adequate countermeasures are not properly taken (e.g., suppression of the TMS click by the use of a masking noise, use of ear defenders, application of a foam layer under the coil) [99,199]. This should lead to careful review of past studies where sensory input by TMS was not properly masked; in particular, it should be noted that effective masking procedures were only seldom used in studies involving older adults. This should prompt to strict control of these confounding factors in future work. 

In conclusion, we believe that TMS and TMS-EEG can give an important contribution to the understanding of the mechanisms underlying physiological brain aging, provided that technical pitfalls and interpretation biases are considered. Future studies should seek to integrate electrophysiological and structural data and clarify how these relate to impairment of daily activities in the elderly population, with the ultimate goal of reliably distinguishing physiological and compensatory processes from disease. 

## Data Availability

Not applicable.
